# Side deep placement of slow-release N as base fertilizer combined with urea as panicle fertilizer increases rice yield by optimizing dry matter and N accumulation and translocation

**DOI:** 10.3389/fpls.2025.1600215

**Published:** 2025-05-29

**Authors:** Mingming Hu, Zhixin Li, Kairui Chen, Ying Xiong, Yongheng Luo, Ailing Wang, Leilei Li, Chuanhai Shu, Zongkui Chen, Zhiyuan Yang, Yongjian Sun, Jun Ma

**Affiliations:** Rice Research Institute of Sichuan Agricultural University/Sichuan Provincial Key Laboratory of Crop Physiology, Ecology and Cultivation, Chengdu, China

**Keywords:** rice, side deep fertilization, slow-release N fertilizer, N use efficiency, yield

## Abstract

**Introduction:**

Side deep fertilization (SDF) technology and slow release N fertilizer offer advantages in enhancing rice yield and N use efficiency. However, the effects of side deep application of slow-release N as base fertilizer, combined with the application of urea at different growth stages, on dry matter accumulation, N uptake, translocation, assimilation, and yield formation in rice remain unclear.

**Methods:**

Therefore, in 2023 and 2024, a field experiment was conducted in Sichuan, China, using the local variety Chuankangyou 6308. Seven N treatments were established: no N (0N), conventional fertilization control (CK), 100% urea side deep placement (DU), 100% slow-release N fertilizer side deep placement (DSU), and DSU combined with 30% urea applied as basal fertilizer (DSU+U), tillering fertilizer (DSU+TU), or panicle fertilizer (DSU+PU).

**Results:**

The results showed that compared with CK, slow-release N fertilizer treatments significantly increased yield by 10.04%~23.28%, with DSU+PU having the highest yield, due to a higher number of effective panicles and seed-setting rate. DSU+PU is beneficial for increasing SPAD value and plant height, significantly increased total dry matter and N accumulation by 24.36% and 34.76%, respectively. The contribution rates of stem and leaf dry matter and N to yield increased by 9.67% and 4.40%, as well as 6.63% and 7.27%, respectively. Nitrate reductase (NR) and glutamine synthetase (GS) activities significantly increased by 4.88% and 6.86%, respectively. Notably, DSU+PU significantly improved N agronomic efficiency (NAE) and N fertilizer partial factor productivity (NPFP) by 89.00% and 23.27%, respectively. Meanwhile, DSU+PU reduced one fertilization application compared with CK and lowered N fertilizer costs compared with DSU.

**Discussion:**

In conclusion, side deep placement of slow-release N as base fertilizer combined with urea as panicle fertilizer can delay leaf senescence, increase plant height and N metabolism enzyme activity, optimize dry matter and N accumulation and distribution, and significantly increase yield, and N use efficiency. It provides a solution for developing a simplified and efficient rice fertilization technology system based on the integration of agricultural machinery and agronomy.

## 1 Introduction

China is a major rice-producing and consuming country, with its rice planting area and total production accounting for 20% and 30% of the global total, respectively (Zhang et al., 2024). Nitrogen is a key nutrient for rice growth and development, contributing to more than 50% of yield formation (Liu et al., 2018). However, China’s N use efficiency is only 30%-35%, significantly lower than the global average (Zhang et al., 2017a). The traditional method of N fertilizer application involves the broadcast application of quick-release urea in multiple splits (3~4 times), which not only results in short fertilizer efficiency and increases labor intensity and costs (Zhong et al., 2020), but also exacerbates N runoff losses and enhances greenhouse gas emissions (Coskun et al., 2017). Against this backdrop, developing mechanized precision fertilization technology has become an inevitable choice for achieving simplified and efficient rice cultivation.

Side deep fertilization (SDF) allows for the precise application of fertilizer granules 2 ~ 5cm to the side and 3~5cm deep into the soil adjacent to rice seedlings during transplanting (Zhong et al., 2019), with the fertilizer being covered by nearby soil, thereby achieving deep placement of N fertilizer (Zhang et al., 2017b). Studies have shown that, under equal N application rates, SDF can increase rice yield by 4.7%~8.0% and improve N recovery efficiency by 21.5%~50.8%. Its efficiency-enhancing mechanism mainly lies in boosting aboveground dry matter accumulation, leaf area index, and net photosynthetic rate at the heading stage (Pan et al., 2017a). Further research has revealed that SDF can also enhance N metabolism by increasing the activities of nitrate reductase (NR) and glutamine synthetase (GS) in the flag leaf. Additionally, the larger leaf area index during the rice heading stage facilitates the accumulation of more photosynthetic products, leading to a yield increase of 11.8%~19.6% (Li et al., 2021). However, some studies have found that SDF only marginally increases yield by 1.1%~1.2% compared with conventional fertilization methods, with a slight improvement in N agronomic efficiency by 1.7%~3.5%, though these differences are not statistically significant, possibly due to a decline in photosynthetic capacity in later growth stages (Zhu et al., 2021). Notably, under high N application rates, SDF may also induce root toxicity effects, leading to excessive vegetative growth, delayed maturity, and a reduced harvest index, which can significantly decrease yield (Chen et al., 2016).

The application of slow-release N fertilizer can effectively mitigate such toxicity effects and is also a viable approach to improving N use efficiency. As a new type of fertilizer, slow-release N fertilizer works by reducing the nutrient release rate, ensuring synchronization between nutrient supply and crop demand (Wang et al., 2016). Studies have confirmed that slow-release N fertilizers optimize N supply dynamics, significantly promote the redistribution of stored substances in rice stems and leaves, and enhance the synthesis of photosynthetic products after heading (Deng et al., 2012). They can increase N fertilizer partial factor productivity by 6%~23%, N agronomic efficiency by 26%~71%, and yield by 8%~19% (Peng et al., 2014). Some studies have also found that compared with conventional fertilization, slow-release N fertilizers increase total soil N and available N content, improve N use efficiency by 44%~53%, and significantly reduce N losses caused by runoff, leaching, and greenhouse gas emissions, ultimately leading to a 7%~12% yield increase (Su et al., 2024). However, the application of slow-release N fertilizers still faces significant technical bottlenecks. First, their nutrient release patterns typically follow a “J”-shaped or “S”-shaped single-peak curve, which can lead to delayed tillering in rice or insufficient nutrient supply at later growth stages (Ke et al., 2018, 2023). Second, the high production cost limits large-scale adoption (Wang et al., 2023). Third, due to their lower density, slow-release N fertilizers tend to float in paddy fields, affecting spatial distribution uniformity and sustained nutrient release efficiency (Ma et al., 2017).

At present, research on slow-release N fertilizers at home and abroad mainly focuses on reduction and efficiency enhancement (Tian et al., 2022), type comparisons (Wu et al., 2021), and optimization of slow-release urea ratios (Cheng et al., 2022). In addition, some scholars have proposed that combining slow-release N fertilizers with urea application can achieve multi-stage N supply, further reducing the cost of slow-release N fertilizers use while ensuring yield. Experimental results have shown that the combined application of slow-release fertilizer and urea (one basal and one tillering application) during the early growth stage of rice not only promotes tiller development but also enhances dry matter conversion and increases the crop growth rate (Xing et al., 2015). Other studies have also found that using slow-release fertilizer as the basal fertilizer and urea as the panicle fertilizer can significantly increase the total leaf area index and the effective leaf area index of rice, enhance N accumulation at the heading and maturity stages in mechanically transplanted hybrid rice, promote the activity of N metabolism enzymes in flag leaves after heading, and thereby improve yield (Lv et al., 2021). Although many studies have independently examined the basic application of slow-release N fertilizers or topdressed urea, studies on side deep application of slow-release N fertilizers as base fertilizer, combined with the application of urea at different growth stages, are rarely reported. Current fertilization methods are still dominated by manual broadcasting, with little emphasis on mechanized precision placement and aligning nutrient supply with the dynamic N demand of crops. Moreover, the comprehensive effects of this integrated fertilization strategy on rice dry matter accumulation, N uptake and translocation, physiological metabolism, and yield formation have not been fully clarified. Therefore, during 2023 and 2024, we conducted a field experiment with rice variety Chuankangyou 6308, a widely promoted variety in the southwestern rice-growing region, investigates the effects of combined application of slow-release N fertilizer and urea under side deep application on SPAD values, dry matter and N accumulation and translocation, N metabolism enzyme activities, N utilization, yield, and yield components in rice. The goal is to establish a simplified and efficient rice fertilization technology system integrating agricultural machinery and agronomy, providing theoretical support and technical guidance for green and high-yield rice cultivation.

## 2 Materials and methods

### 2.1 Experimental site and materials

**Figure 1:**
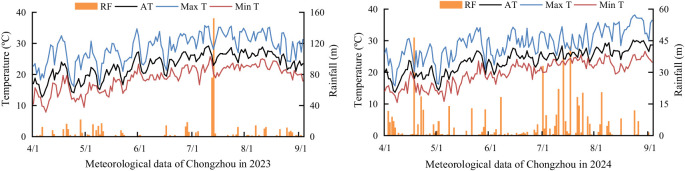
Meteorological data of rice season in Chongzhou from 2023 to 2024. RF, Rainfall; AT, Average temperature; Max T, Maximum temperature; Min T, Minimum temperature.

The field experiment was conducted from 2023 to 2024 at the Modern Agricultural Research Park of Sichuan Agricultural University (30°42′ N, 103°28′ E)in Chongzhou, Sichuan, China. The experimental site has a subtropical monsoon climate and was fallow prior to the trial. Meteorological data for the rice-growing season were provided by the Sichuan Meteorological Bureau, as shown in **Figure 1**. The topsoil (0~20 cm) is sandy loam, and its basic physicochemical properties are listed in **Table 1**. The tested rice variety was Chuankangyou 6308, a major hybrid indica rice variety in the southwestern rice-growing region of China, with a full growth period of 153~156 days.

**Table 1:** Basic soil physical and chemical properties.

Year	pH	Organic Matter (g kg^-1^)	Total N (g kg^-1^)	NH_4_^+^-N (mg kg^-1^)	NO_3_^–^N (mg kg^-1^)	Total P (g kg^-1^)	Total K (g kg^-1^)	Available P (mg kg^-1^)	Available K (mg kg^-1^)
2023	7.01	25.47	1.87	37.81	5.53	0.71	4.81	28.47	236.93
2024	6.93	24.74	1.72	33.76	5.34	0.63	5.17	21.77	226.63

### 2.2 Experimental design

The experiment was conducted using a single-factor randomized block design with seven pure N treatments, which were as follows: no N (0N), conventional fertilization control (CK), 100% urea as basal fertilizer side deep placement (DU), 100% slow-release N fertilizer as basal fertilizer side deep placement (DSU), and DSU combined with 30% urea applied as basal fertilizer (DSU+U), tillering fertilizer (DSU+TU), or panicle fertilizer (DSU+PU). The total N application rate for all fertilization treatments was 150 kg ha^-1^. In the experiment, the N basal fertilizer was side deep applied (about 3~5 cm) on the same day as mechanical transplanting, the tillering fertilizer was manually broadcasted 10 days after transplanting, and the panicle fertilizer was broadcasted manually at the fourth-leaf stage. Specific experimental treatments are shown in **Table 2**. The slow-release N fertilizer used was resin-coated slow-release N fertilizer, provided by Hebei Dewoduo Fertilizer Co., Ltd., with 100% coating and a N content of 44.0%, theoretical release period is 90 days. Regular urea had a N content of 46%, potassium fertilizer was potassium chloride (K_2_O≥60%, 150 kg ha^-1^), and phosphorus fertilizer was calcium superphosphate (P_2_O_5_≥12%, 75 kg ha^-1^). All phosphorus and potassium fertilizers were applied one day before mechanical transplanting. The plot area was 5 m ×3.8 m=19 m², with three replicates, totaling 21 plots. The planting density was 30 cm×18 cm between rows and holes, with 3~5 seedlings per hole. Each plot was leveled and separated by plastic film to ensure independent irrigation and drainage. Seedlings were raised using blanket sowing, with 80 g of dry seeds per tray. In 2023, seeds were sown on April 3, mechanical transplanting occurred on April 25, and harvesting was on September 2. In 2024, seeds were sown on March 23, mechanical transplanting occurred on April 20, and harvesting was on August 26. The water management regime involved maintaining soil moisture at transplanting, intermittent irrigation during tillering, natural water cutoff when the number of stems reached 80% of the expected yield, and intermittent irrigation from jointing to maturity. Pest, disease, and weed control, as well as other management practices, were carried out according to the local large-scale production standards.

**Table 2:** Experiment treatments.

Trement	Base Fertilizer		Tillering Fertilizer (urea)	Panicle Fertilizer (urea)	Total N (kg ha^-1^)
	Slow-Release Urea	Urea			
0N	0	0	0	0	0
CK	0	40%	30%	30%	150
DU	0	100%	0	0	150
DSU	100%	0	0	0	150
DSU+U	70%	30%	0	0	150
DSU+TU	70%	0	30%	0	150
DSU+PU	70%	0	0	30%	150

0N, No N; CK, conventional fertilization control; DU, 100% urea side deep placement; DSU, 100% slow-release N fertilizer side deep placement; DSU+U, DSU combined with 30% urea applied as basal fertilizer side deep placement; DSU+TU, DSU combined with 30% urea applied as tillering fertilizer; DSU+PU, DSU combined with 30% urea applied as panicle fertilizer.

### 2.3 Sampling and measurements

#### 2.3.1 Slow-release N fertilizers release rate

On the day of rice transplanting, 10 g of resin-coated slow-release N fertilizer was weighed and placed in nylon mesh bags, which were evenly buried at a depth of 5 ~ 7 cm in the soil between rice rows. Samples were collected every 10 days, for a total of 10 sampling times. The collected fertilizer samples were cleaned, completely dried in a 40°C oven, and the nutrient release rate of the slow-release N fertilizer was calculated using the mass difference method.

#### 2.3.2 Grain yield and yield components

At the maturity stage, 30 hills were selected from each plot to investigate the number of effective panicles. Based on the average number of effective panicles, 5 hills were selected from each plot for grain analysis, measuring the number of filled and unfilled grains, seed-setting rate, and thousand-grain weight. The actual grain yield was measured when the moisture content of the rice grains reached 13.5%.

#### 2.3.3 SPAD values and plant height

At the peak tillering stage (31 days after transplantation), jointing stage (57 days after transplantation), heading stage (97 days after transplantation), and maturity stage (126 days after transplantation), 10 representative plants were selected from each plot. A chlorophyll meter (Minolta, Japan) was used to measure the SPAD value at three points (upper, middle, and lower one-third) of the last fully expanded leaf, avoiding the leaf veins. The average value was recorded. The plant height was measured from the soil surface to the tip of the longest leaf.

#### 2.3.4 Dry matter accumulation and translocation

At the peak tillering stage (31 days after transplantation), jointing stage (57 days after transplantation), heading stage (97 days after transplantation), and maturity stage (126 days after transplantation), representative plants from three hills were selected from each plot based on the average number of culms and tillers. The plants were separated into stems, leaves, and panicles (at heading and maturity stages). The samples were inactivated at 105°C for 30 minutes, then dried at 80°C to a constant weight. The dry matter of each organ was measured to calculate dry matter accumulation and translocation.

1. Stem dry matter translocation (kg ha^-1^) = Dry matter of stem at heading stage − Dry matter of stem at maturity stage
2. Leaf dry matter translocation (kg ha^-1^) = Dry matter of leaves at heading stage − Dry matter of leaves at maturity stage
3. Stem dry matter translocation rate (%) = (Stem dry matter translocation/Dry matter of stem at heading stage) × 100
4. Leaf dry matter translocation rate (%) = (Leaf dry matter translocation/Dry matter of leaves at heading stage) × 100
5. Contribution rate of stem dry matter translocation (%) = (Stem dry matter translocation/Dry matter of panicle at maturity stage) × 100
6. Contribution rate of leaf dry matter translocation (%) = (Leaf dry matter translocation/Dry matter of panicle at maturity stage) × 100

#### 2.3.5 N accumulation and translocation

Samples from 1.3.4 were ground and sieved, then digested using concentrated H_2_SO_4_-H_2_O. The total N content of each organ was determined by the Kjeldahl method (Kjeldahl 8400, FOSS, Denmark), and N accumulation and translocation were calculated.

1. Stem N translocation (kg ha^-1^) = N accumulation in stem at heading stage − N accumulation in stem at maturity stage
2. Leaf N translocation (kg ha^-1^) = N accumulation in leaves at heading stage − N accumulation in leaves at maturity stage
3. Stem N translocation rate (%) = (Stem N translocation/N accumulation in stem at heading stage) × 100
4. Leaf N translocation rate (%) = (Leaf N translocation/N accumulation in leaves at heading stage) × 100
5. Contribution rate of stem N translocation (%) = (Stem N translocation/N accumulation in panicle at maturity stage) × 100
6. Contribution rate of leaf N translocation (%) = (Leaf N translocation/N accumulation in panicle at maturity stage) × 100

#### 2.3.6 N metabolism enzyme activity

In 2023, at the heading stage of rice, 60 uniform and healthy plants were selected from each treatment and tagged. Samples were collected on sunny days between 9:30 and 10:30. Tagged plants were selected at 7, 14, 21, and 28 days after heading, with 10 plants sampled each time. The flag leaves were harvested, immediately fixed in liquid N, and stored in a cryogenic freezer (-80°C) for later analysis. The activities of nitrate reductase (NR) and glutamine synthetase (GS) were measured using biochemical reagent kits (purchased from Jiangsu Enzyme Immuno Industry Co., Ltd.), with three replications. Measurement of NR and GS Activities by Double-Antibody Sandwich ELISA. A 1 g sample of plant tissue was accurately weighed and homogenized in 9 mL of phosphate-buffered saline (PBS) with a pH of approximately 7.2-7.4 using a homogenizer. The homogenate was centrifuged at 2000–3000 r min^-1^ for 20 minutes, and the supernatant was collected for analysis. Blank wells, standard wells, and sample wells were prepared separately. In the ELISA plate, 50 μL of standard solution was added to each standard well. For the sample wells, 40 μL of sample dilution buffer was added first, followed by 10 μL of the sample. The plate was sealed with a cover membrane and incubated at 37°C for 30 minutes. After incubation, the 30× concentrated wash solution was diluted with distilled water at a ratio of 1:30 for later use. The membrane was carefully removed, and the liquid was discarded by flicking. Each well was filled with wash buffer, allowed to stand for 30 seconds, and the buffer was discarded. This washing step was repeated five times, and the plate was blotted dry. Next, 50 μL of enzyme-labeled reagent was added to each well except the blank wells. After a second incubation and washing process (following the same procedure as above), 50 μL of Color Developer A and 50 μL of Color Developer B were sequentially added to each well. The plate was gently mixed and incubated at 37°C in the dark for 10 minutes for color development. Then, 50 μL of stop solution was added to each well to terminate the reaction (the color changed from blue to yellow). The absorbance (OD value) was measured sequentially at 450 nm, using the blank wells as a zero reference.

#### 2.3.7 N use efficiency

The N agronomic efficiency (NAE) and N fertilizer partial factor productivity (NPFP)were calculated based on yield at the maturity stage:

1. NAE (kg kg^-1^) = (Yield in the N-treated plot−Yield in the no-N plot)/N application rate
2. NPFP (kg kg^-1^) = Yield in the N-treated plot/N application rate

### 2.4 Statistical analysis

Data were statistically processed using Microsoft Excel 2016 (Microsoft Corp., Redmond, WA, USA) and analyzed with SPSS 27.0 (IBM, Inc., Armonk, NY, USA). Figures were plotted using Origin 2024 (OriginLab, Northampton, MA, USA). Significant differences were determined using the Least Significant Difference (LSD) method at the p< 0.05 level.

## 3 Results

### 3.1 Slow-release N fertilizers release rate

**Figure 2:**
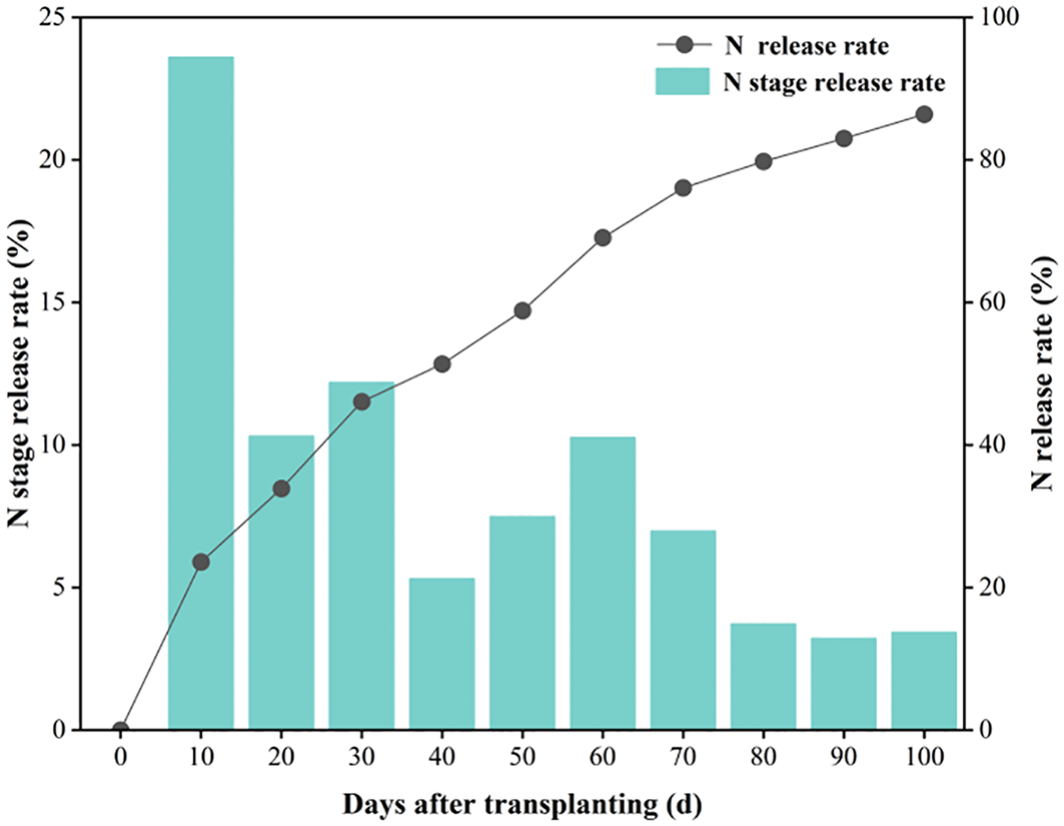
Slow-release N fertilizers release rate.

The nutrient release of slow-release N fertilizer follows a “rapid release in the early stage - stable release in the middle stage - slow release in the later stage” pattern (**Figure 2**). Within 100 days, the release completion rate is approximately 86.41%. During the initial 0–10 days, the release rate is relatively fast, with a release rate of 23.59%. Then, from days 10 to 70, the release enters a steady phase, with nutrients released at a relatively uniform rate, reaching a release completion rate of 76.07%. Finally, during the 70–100 days period, the release rate significantly slows down, accounting for 10.34% of the total release.

### 3.2 Grain yield and yield components

Variance analysis showed that the year had a highly significant effect on the number of effective panicles, seed-setting rate, and 1000-grain weight, while N management had a highly significant effect on all indicators. The interaction between the two had a significant or highly significant effect on the number of spikelets per panicle, seed-setting rate, and 1000-grain weight (**Table 3**). Compared with 0N, N application for two years significantly increased yield by 35.61%~67.15% (**Table 4**). Compared with CK, all side deep fertilization treatments were beneficial for increasing rice yield. Among them, DU increased the yield by 2.58% compared with CK over two years, but the difference was not significant. Slow-release N fertilizer treatments significantly increased the yield by 10.04%~23.28% compared with CK over two years, with DSU+PU showing the highest yield. Compared with DSU, the combined application of slow-release N fertilizer and urea treatments significantly increased the yield by 5.05%~12.04% over two years. Among them, DSU+PU’s yield was significantly higher than DSU+U and DSU+TU, increasing by 5.18% and 6.66%, respectively, over two years. The yield of DSU+U was slightly higher than DSU+TU, but the difference was not significant.

**Table 3:** Variance analysis of rice photosynthetic material production, N accumulation and transport, and yield in response to year and N treatment.

Index		Year(Y)	N treatment (N)	Y×N
SPAD	Peak tillering stage	11.37**	42.94**	0.28ns
	Jointing stage	16.95**	79.12**	22.60**
	Heading stage	131.46**	60.67**	4.72**
	Maturity stage	1382.34**	127.91**	3.98**
Plant height	Peak tillering stage	5.54*	48.65**	0.08ns
	Jointing stage	15.33**	105.88**	6.99**
	Heading stage	0.15ns	63.60**	1.34**
	Maturity stage	11.75**	78.99**	4.27**
Total dry matter accumulation	Peak tillering stage	9.19**	388.73**	2.83*
	Jointing stage	16.08**	426.14**	13.19**
	Heading stage	560.93**	1117.68**	38.77**
	Maturity stage	176.75**	1265.28**	23.10**
Total N accumulation	Peak tillering stage	27.04**	507.47**	2.40ns
	Jointing stage	0.01ns	598.31**	12.71**
	Heading stage	109.05**	1477.29**	38.22**
	Maturity stage	490.53**	3009.47**	94.02**
Stem	Dry matter translocation amount	2112.62**	438.76**	67.02**
	Dry matter output rate	1322.63**	127.62**	26.72**
	Dry matter contribution rate	1501.48**	138.50**	33.25**
	N translocation amount	599.45**	465.56**	30.75**
	N output rate	951.79**	283.32**	23.80**
	N contribution rate	828.89**	205.96**	20.74**
Leaf	Dry matter translocation amount	293.98**	75.45**	4.54**
	Dry matter output rate	264.37**	13.71**	3.33*
	Dry matter contribution rate	386.64**	54.59**	2.93*
	N translocation amount	73.46**	428.90**	10.37**
	N output rate	326.21**	40.87**	20.89**
	N contribution rate	2.09ns	87.87**	4.37**
N agronomic efficiency		5.50*	57.20**	0.08ns
N fertilizer partial factor productivity		2.92ns	57.24**	0.08ns
Effective panicles		39.03**	142.67**	0.72ns
Spikelets per panicle		0.02ns	1725.31**	59.39**
Seed setting rate		32242.93**	8682.88**	556.77**
1000-grain weight		225.62**	68.54**	2.62*
Yield		2.25ns	197.04**	0.21ns

* and ** indicate significant at 5% and 1% levels, respectively, and ns indicates no significant difference.

**Table 4:** Yield and yield components of rice for different N treatments in 2023 and 2024.

Year	Treatment	Effective Panicles(×10^4^ ha^-1^)	Spikelets per Panicle	Seed Setting rate (%)	1000-Grain Weight (g)	Yield(kg ha^-1^)
2023	0N	234.82 ± 10.92e	131.92 ± 0.45f	83.60 ± 0.05a	25.84 ± 0.11c	6690.76 ± 311.06e
	CK	285.73 ± 6.78d	153.79 ± 0.05c	75.35 ± 0.01e	27.01 ± 0.01a	8941.94 ± 212.26d
	DU	305.43 ± 18.96c	145.86 ± 0.44d	77.77 ± 0.07c	26.58 ± 0.10b	9207.97 ± 571.67d
	DSU	307.08 ± 9.75c	153.71 ± 0.46c	77.78 ± 0.06c	26.88 ± 0.10a	9868.43 ± 108.79c
	DSU+U	367.83 ± 9.43a	143.67 ± 0.40e	75.09 ± 0.07f	26.53 ± 0.10b	10526.17 ± 269.91b
	DSU+TU	328.42 ± 9.76b	154.64 ± 0.05b	76.73 ± 0.01d	26.47 ± 0.01b	10317.11 ± 306.66bc
	DSU+PU	321.85 ± 6.85bc	160.01 ± 0.17a	79.77 ± 0.02b	27.02 ± 0.03a	11100.66 ± 236.33a
2024	0N	215.11 ± 4.52e	137.47 ± 0.66g	85.15 ± 0.07a	26.35 ± 0.15c	6635.04 ± 139.35e
	CK	270.89 ± 4.44d	156.81 ± 0.99b	78.83 ± 0.14f	27.25 ± 0.22ab	9127.01 ± 149.72d
	DU	293.39 ± 12.97c	145.11 ± 0.44e	80.55 ± 0.06e	27.20 ± 0.10b	9325.99 ± 412.40d
	DSU	297.22 ± 3.04c	150.22 ± 0.85d	81.96 ± 0.11c	27.36 ± 0.19ab	10013.12 ± 102.45c
	DSU+U	341.56 ± 12.07a	141.91 ± 0.43f	80.73 ± 0.06d	27.22 ± 0.10b	10651.63 ± 376.52b
	DSU+TU	316.93 ± 13.92b	152.54 ± 0.43c	80.59 ± 0.06de	27.13 ± 0.10b	10567.65 ± 464.06b
	DSU+PU	304.34 ± 3.77bc	159.36 ± 0.60a	83.77 ± 0.06b	27.50 ± 0.12a	11172.37 ± 138.30a

0N, No N; CK, conventional fertilization control; DU, 100% urea side deep placement; DSU, 100% slow-release N fertilizer side deep placement; DSU+U, DSU combined with 30% urea applied as basal fertilizer side deep placement; DSU+TU, DSU combined with 30% urea applied as tillering fertilizer; DSU+PU, DSU combined with 30% urea applied as panicle fertilizer. Different lowercase letters indicate significant differences between N treatments in the same year at the p<0.05 level.

Regarding yield components, compared with CK, all side deep fertilization treatments significantly increased the number of effective panicles, with a 2-year increase of 7.60%~27.41%, with DSU+U showing the highest increase. The number of spikelets per panicle showed an overall decreasing trend, with significant differences between treatments, but DSU+PU was significantly higher than CK, with a 2-year increase of 2.84%. The seed-setting rate also showed a significant increasing trend, with a 2-year increase of 0.92%~6.07%, with DSU+PU showing the highest increase. 1000-grain weight was highest in DSU+PU, but there was no significant difference between DSU, CK, and DSU+PU.

### 3.3 SPAD values

**Figure 3:**
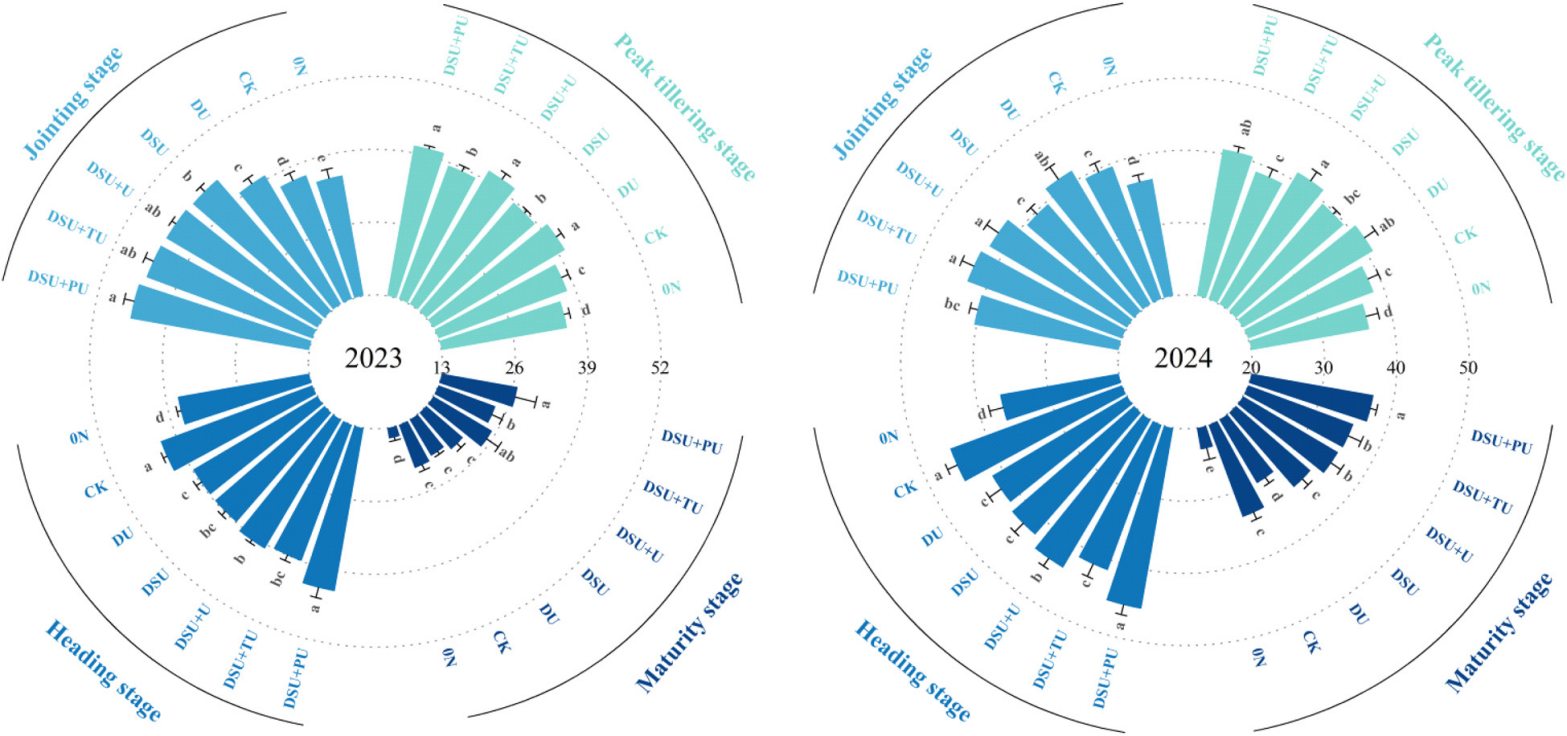
Flag leaf SPAD values of rice for different N treatments in 2023 and 2024. 0N, No N; CK, conventional fertilization control; DU, 100% urea side deep placement; DSU, 100% slow-release N fertilizer side deep placement; DSU+U, DSU combined with 30% urea applied as basal fertilizer side deep placement; DSU+TU, DSU combined with 30% urea applied as tillering fertilizer; DSU+PU, DSU combined with 30% urea applied as panicle fertilizer. Different lowercase letters indicate significant differences in N treatment at the p<0.05 level during the same growth stage.

Variance analysis showed that the year, N management, and their interaction (except for the peak tillering stage) had highly significant effects on the SPAD values of leaves at different growth stages (**Table 3**). At the peak tillering stage, the SPAD values of all side deep fertilization treatments were significantly higher than CK, with a 2-year increase of 1.64%~7.85%, with DSU+U showing the highest values, but no significant differences were found between DU, DSU+PU, and CK (**Figure 3**). At the jointing stage, in 2023, the SPAD values of all side deep fertilization treatments were significantly higher than CK, increasing by 8.57%~24.00%. The SPAD values of slow-release fertilizer treatments were also significantly higher than DU, increasing by 10.99%~14.21%, with DSU+PU showing the highest value, but no significant difference was observed between DSU+U, DSU+TU, and DU. In 2024, the SPAD values of all side deep fertilization treatments were higher than CK, but no significant difference was found between DSU, DSU+PU, and CK, with DSU+TU showing the highest value.

At the heading and maturity stages, the SPAD values of the flag leaf were highest in DSU+PU, with DSU+PU at heading showing significantly higher values than the other side deep fertilization treatments, with a 2-year increase of 5.37%~9.76%, but no significant difference was observed compared with CK. At maturity, the SPAD values of all slow-release fertilizer treatments were higher than CK, with no significant difference between DSU and CK. The combined application of slow-release N fertilizer and urea treatments significantly increased the SPAD value by 8.64%~18.34% compared with CK over two years.

### 3.4 Plant height

**Figure 4:**
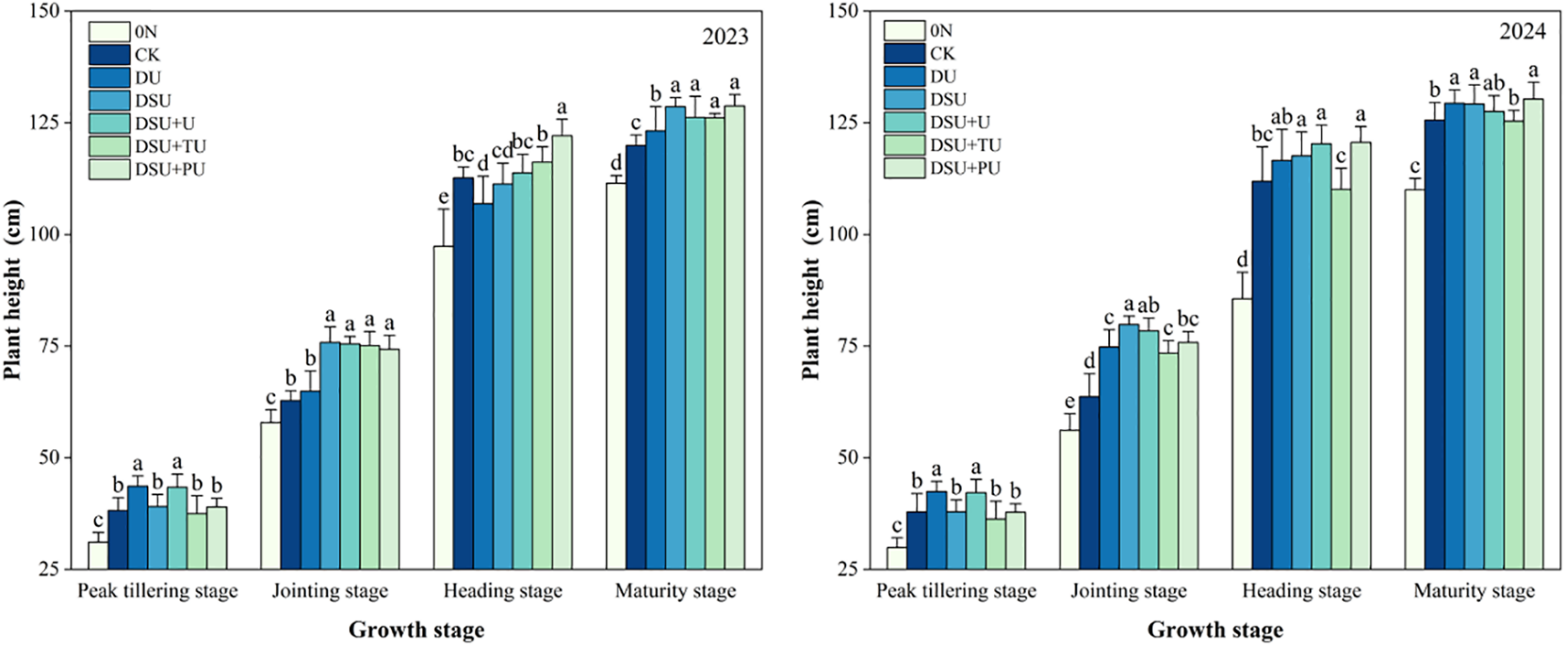
Plant height of rice for different N treatments in 2023 and 2024. 0N, No N; CK, conventional fertilization control; DU, 100% urea side deep placement; DSU, 100% slow-release N fertilizer side deep placement; DSU+U, DSU combined with 30% urea applied as basal fertilizer side deep placement; DSU+TU, DSU combined with 30% urea applied as tillering fertilizer; DSU+PU, DSU combined with 30% urea applied as panicle fertilizer. Different lowercase letters indicate significant differences in N treatment at the p<0.05 level during the same growth stage.

Analysis of variance showed that the year (except at the heading stage), N management, and their interaction (except at the peak tillering stage) had significant or highly significant effects on plant height at different growth stages (**Table 3**). At the peak tillering stage, the plant height of all side-deep fertilization treatments was generally higher than that of CK, with DU and DSU+U showing significant differences compared with CK (**Figure 4**). At the jointing stage, plant height under all side-deep fertilization treatments was significantly increased by 9.29%~22.99% compared with CK, with the highest value observed in DSU.

At the heading and maturity stages, DSU+PU consistently resulted in the greatest plant height, showing significant increases of 8.06% and 5.58% over CK, respectively. Differences among the slow-release fertilizer treatments were mostly not significant.

### 3.5 Dry matter accumulation

**Figure 5:**
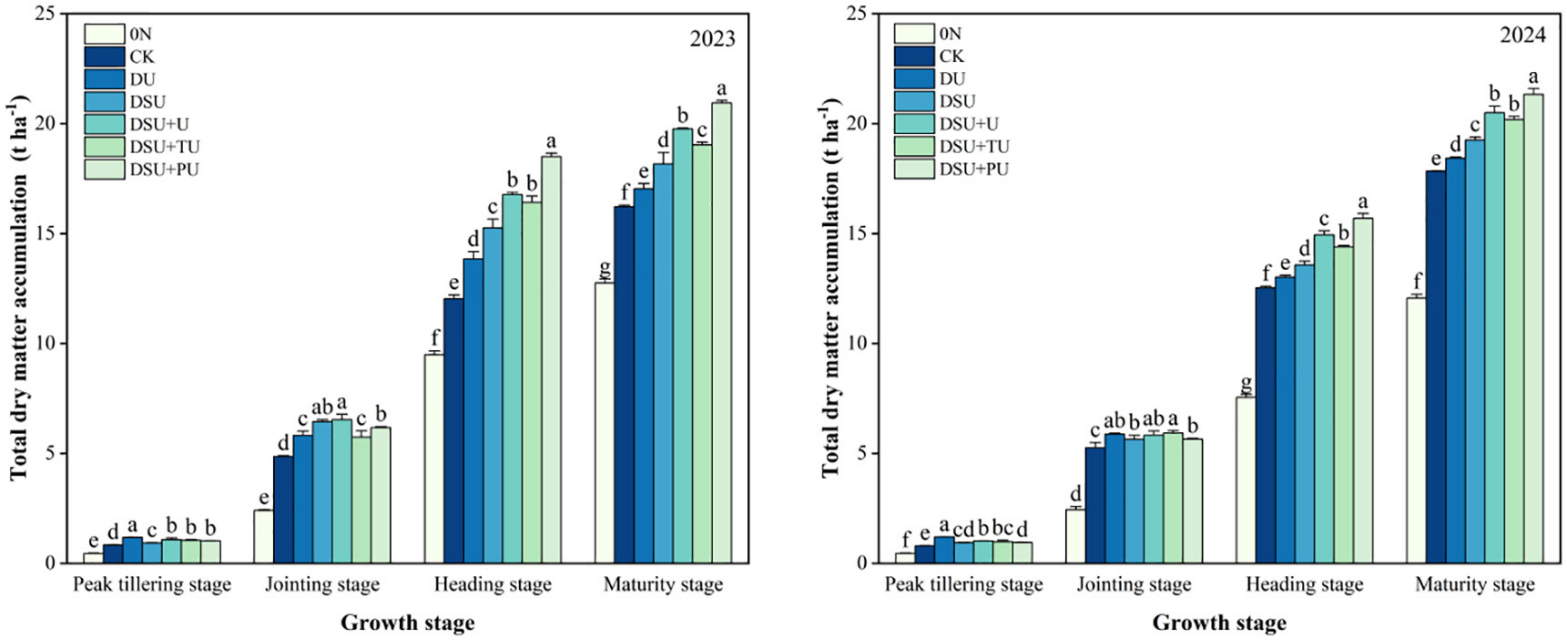
Total dry matter accumulation of rice for different N treatments in 2023 and 2024. 0N, No N; CK, conventional fertilization control; DU, 100% urea side deep placement; DSU, 100% slow-release N fertilizer side deep placement; DSU+U, DSU combined with 30% urea applied as basal fertilizer side deep placement; DSU+TU, DSU combined with 30% urea applied as tillering fertilizer; DSU+PU, DSU combined with 30% urea applied as panicle fertilizer. Different lowercase letters indicate significant differences in N treatment at the p<0.05 level during the same growth stage.

Variance analysis showed that the year, N management, and their interaction had significant or highly significant effects on dry matter accumulation at different growth stages (**Table 3**). At the peak tillering stage, the total dry matter accumulation of all side deep fertilization treatments was significantly higher than that of CK, with a 2-year increase of 14.11%~46.46%, with DU showing the highest accumulation (**Figure 5**). The total dry matter accumulation of DU was significantly higher than that of the slow-release fertilizer treatments, with a 2-year increase of 14.41%~28.34%. Among the slow-release fertilizer treatments, DSU+U had 12.32% higher dry matter accumulation than DSU, with a significant difference between them. At the jointing stage, the total dry matter accumulation of all side deep fertilization treatments was also significantly higher than CK, with a 2-year increase of 12.57%~23.67%, with DSU+U showing the highest accumulation. However, there were mostly no significant differences among the side deep fertilization treatments.

At the heading and maturity stages, the total dry matter accumulation of all side deep fertilization treatments was significantly higher than CK, with a 2-year increase of 9.43%~39.38% and 4.19%~24.36%, respectively, with DSU+PU showing the highest accumulation, significantly differing from other treatments. The total dry matter accumulation of all slow-release fertilizer treatments was significantly higher than DU, with a 2-year increase of 7.24%~27.04% at the heading stage and 11.19%~19.35% at maturity. The total dry matter accumulation of combined application of slow-release N fertilizer and urea treatments was significantly higher than DSU, with a 2-year increase of 6.78%~18.39% at the heading stage and 5.61%~13.06% at maturity.

### 3.6 Dry matter translocation

**Figure 6:**
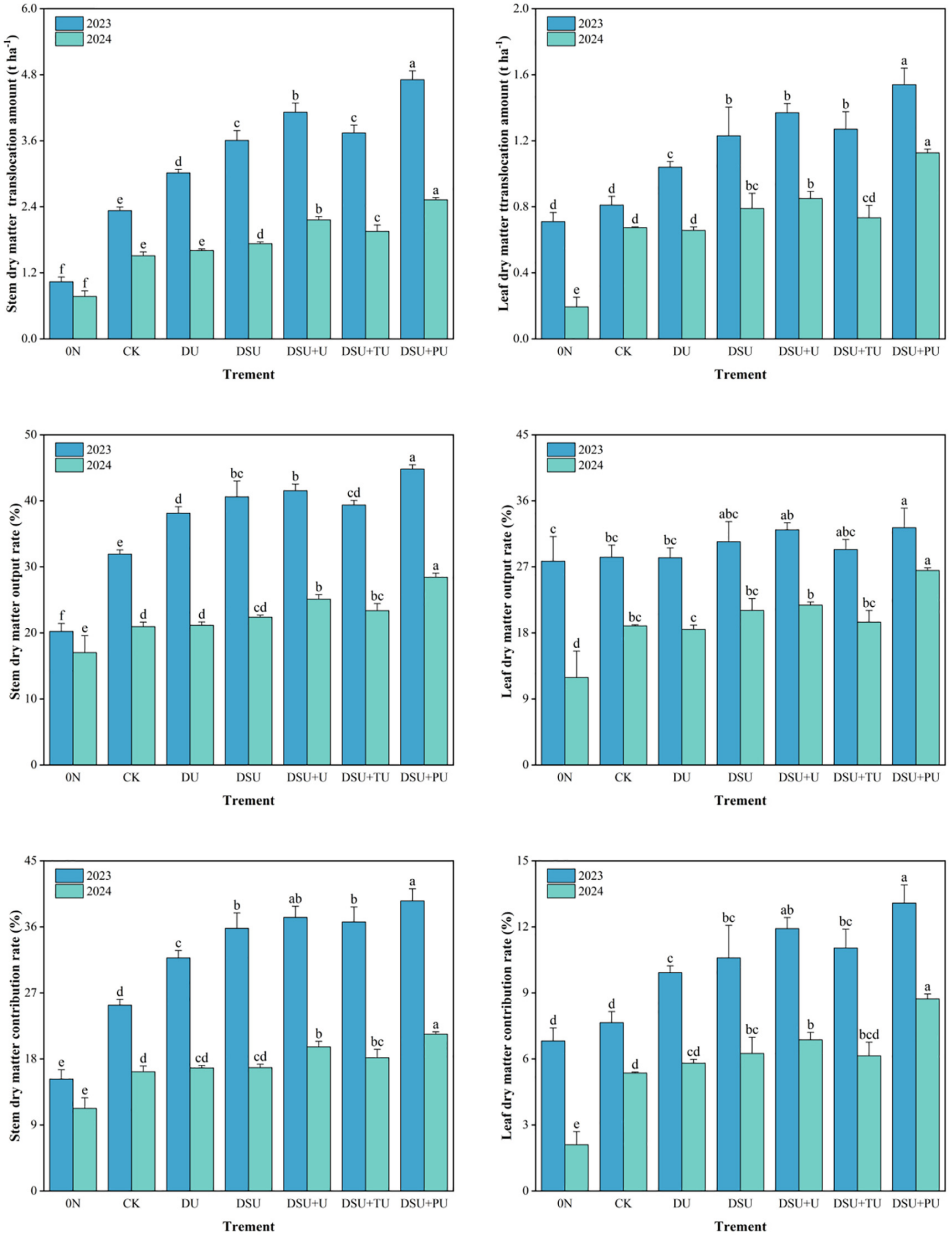
Dry matter translocation of rice for different N treatments in 2023 and 2024. 0N, No N; CK, conventional fertilization control; DU, 100% urea side deep placement; DSU, 100% slow-release N fertilizer side deep placement; DSU+U, DSU combined with 30% urea applied as basal fertilizer side deep placement; DSU+TU, DSU combined with 30% urea applied as tillering fertilizer; DSU+PU, DSU combined with 30% urea applied as panicle fertilizer. Different lowercase letters indicate significant differences between N treatments in the same year at the p<0.05 level.

Analysis of variance indicated that the year, N management, and their interaction had significant or highly significant effects on stem and leaf dry matter translocation (**Table 3**). Under different N treatments, the dry matter translocation amount, output rate, and contribution rate followed the order: stem > leaf (**Figure 6**). Compared with CK, all side deep fertilization treatments promoted stem dry matter translocation, output rate, and contribution rate, with increases of 17.57%~84.85%, 3.20%~10.17%, and 3.49%~9.67% over two years, respectively, with DSU+PU being the highest. Among them, the combined application of slow-release N fertilizer and urea treatments showed significant differences from CK, whereas DU did not show a significant difference from CK in 2024. Compared with DSU, the combined application of slow-release N fertilizer and urea treatments improved stem dry matter translocation, with DSU+PU showing significantly higher translocation amount, output rate, and contribution rate than DSU by 38.36%, 5.13%, and 4.15%, respectively.

Similarly, compared with CK, all side deep fertilization treatments also increased leaf dry matter translocation amount, output rate, and contribution rate, with increases of 13.46%~79.39%, 2.10%~5.78% (except DU), and 1.36%~4.40% over two years, respectively. DSU+PU exhibited the highest values and showed significant differences from most other treatments, whereas DU did not significantly differ from CK. Compared with DSU, the combined application of slow-release N fertilizer and urea treatments (except DSU+TU) enhanced leaf dry matter translocation, with DSU+PU significantly increasing translocation amount, output rate, and contribution rate by 34.12%, 3.68%, and 2.49%, respectively, over DSU. However, DSU did not significantly differ from DSU+U and DSU+TU.

### 3.7 N accumulation

**Figure 7:**
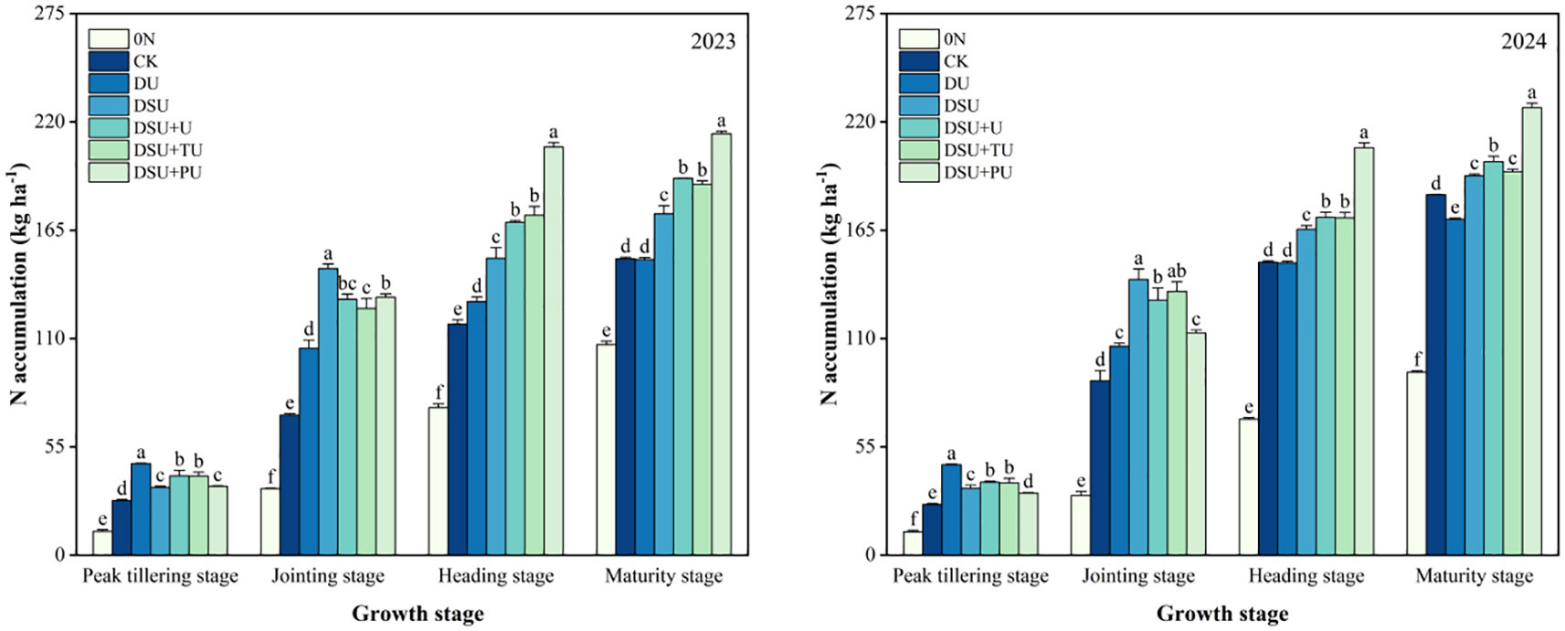
Total N accumulation of rice for different N treatments in 2023 and 2024. 0N, No N; CK, conventional fertilization control; DU, 100% urea side deep placement; DSU, 100% slow-release N fertilizer side deep placement; DSU+U, DSU combined with 30% urea applied as basal fertilizer side deep placement; DSU+TU, DSU combined with 30% urea applied as tillering fertilizer; DSU+PU, DSU combined with 30% urea applied as panicle fertilizer. Different lowercase letters indicate significant differences in N treatment at the p<0.05 level during the same growth stage.

Analysis of variance indicated that the year (except for N accumulation at the jointing stage), N management, and their interaction (except for N accumulation at the peak tillering stage) had significant or highly significant effects on nitrogen accumulation at different growth stages (**Table 3**). At the peak tillering stage, all side deep fertilization treatments significantly increased total N accumulation compared with CK, with increases of 23.19%~73.23% over two years, and DU showed the highest accumulation (**Figure 7**). DU also had significantly higher N accumulation than the slow-release fertilizer treatments, with increases of 19.81%~40.96% over two years. Among the slow-release fertilizer treatments, DSU+U significantly increased N accumulation by 13.09% compared with DSU, although the difference between DSU+U and DSU+TU was not significant. At the jointing stage, all side deep fertilization treatments significantly increased total N accumulation compared with CK, with increases of 33.78%~81.45% over two years, with DSU showing the highest accumulation, significantly differing from other treatments. Additionally, all slow-release fertilizer treatments had significantly higher N accumulation than DU, with increases of 12.84%~35.30% over two years.

At the heading and maturity stages, all slow-release fertilizer treatments significantly increased N accumulation compared with CK, with increases of 19.93%~57.95% and 11.54%~34.76% over two years, respectively, with DSU+PU showing the highest accumulation and significant differences from other treatments. In contrast, DU did not show significant differences from CK in most cases. Among the combined application of slow-release N fertilizer and urea treatments, total N accumulation was significantly higher than DSU, with increases of 7.87%~31.25% at the heading stage and 4.89%~20.73% at the maturity stage; however, there was no significant difference between DSU+U and DSU+TU.

### 3.8 N translocation

**Figure 8:**
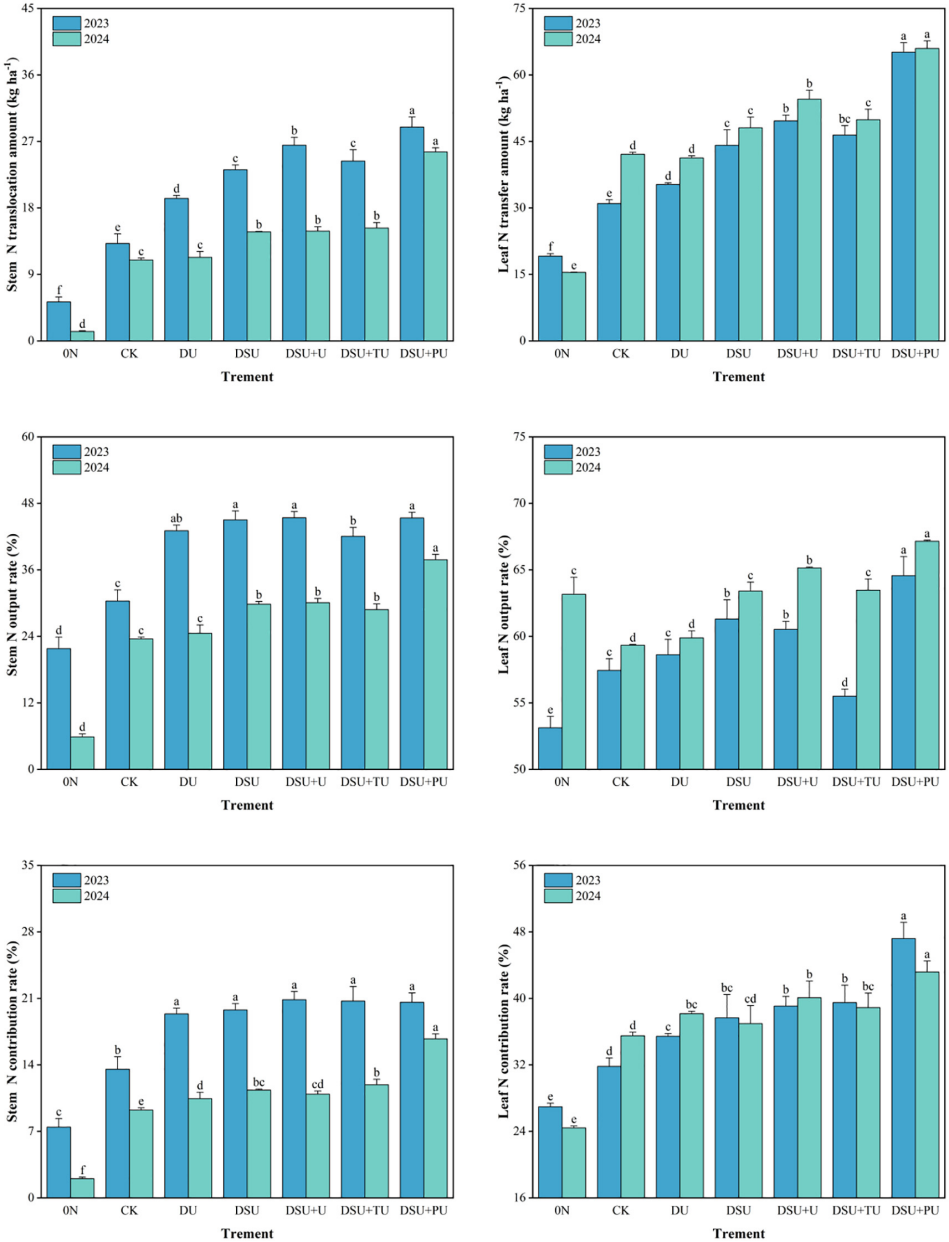
N translocation of rice for different N treatments in 2023 and 2024. 0N, No N; CK, conventional fertilization control; DU, 100% urea side deep placement; DSU, 100% slow-release N fertilizer side deep placement; DSU+U, DSU combined with 30% urea applied as basal fertilizer side deep placement; DSU+TU, DSU combined with 30% urea applied as tillering fertilizer; DSU+PU, DSU combined with 30% urea applied as panicle fertilizer. Different lowercase letters indicate significant differences between N treatments in the same year at the p<0.05 level.

Analysis of variance showed that the year (except for leaf N contribution rate), N management, and their interaction had highly significant effects on stem and leaf N translocation (**Table 3**). Under different N treatments, the N translocation amount, output rate, and contribution rate in rice were higher in leaves than in stems (**Figure 8**). Compared with CK, all side deep fertilization treatments significantly increased stem N translocation amount, output rate, and contribution rate, with increases of 18.20%~111.66%, 4.64%~12.95%, and 2.73%~6.63%, respectively, with DSU+PU showing the highest values. Among them, the combined application of slow-release N fertilizer and urea treatments showed significant differences from CK, while DU did not show significant differences from CK in 2024. Compared with DSU, the combined application of slow-release N fertilizer and urea treatments improved stem N translocation, with DSU+PU showing a significant increase in stem N translocation amount, output rate, and contribution rate by 49.14%, 4.18%, and 3.11%, respectively.

Compared with CK, all side deep fertilization treatments also increased leaf N translocation amount, output rate, and contribution rate, with increases of 5.57%~75.24%, 0.59%~5.56%, and 1.98%~7.27%, respectively, with DSU+PU showing the highest values and significant differences from most other treatments. Compared with DSU, the combined application of slow-release N fertilizer and urea treatments also improved leaf N translocation, with DSU+PU showing a significant increase in leaf N translocation amount, output rate, and contribution rate by 37.13%, 1.73%, and 5.81%, respectively. There were no significant differences between DSU and DSU+U or DSU+TU in most cases.

### 3.9 N metabolism and N use efficiency

**Figure 9:**
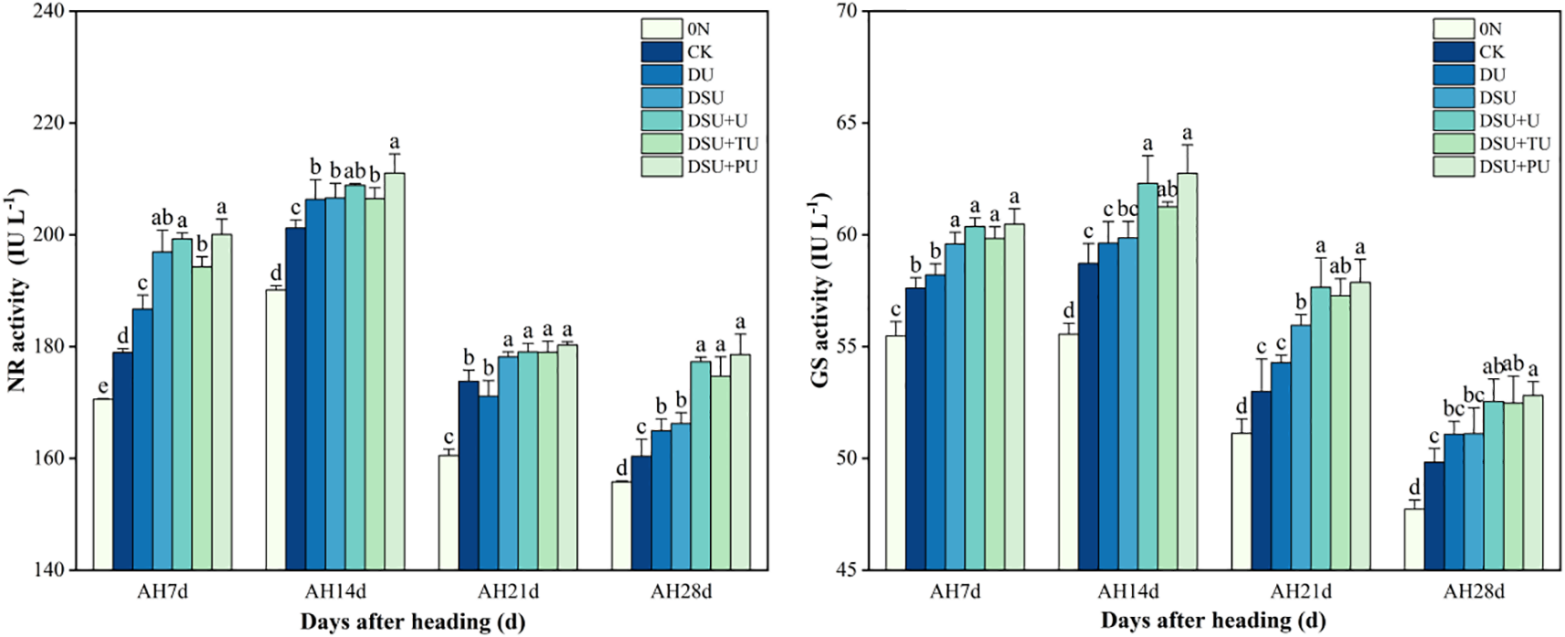
N metabolism enzyme activities of rice for different N treatments in 2023. 0N, No N; CK, conventional fertilization control; DU, 100% urea side deep placement; DSU, 100% slow-release N fertilizer side deep placement; DSU+U, DSU combined with 30% urea applied as basal fertilizer side deep placement; DSU+TU, DSU combined with 30% urea applied as tillering fertilizer; DSU+PU, DSU combined with 30% urea applied as panicle fertilizer. Different lowercase letters indicate significant differences between N treatments at the p<0.05 level during the same period.

Over time, the N metabolism enzyme activity in flag leaves after heading exhibited a trend of first increasing and then decreasing, reaching its peak on the 14th day after heading (**Figure 9**). The N metabolism enzyme activity in all side deep fertilization treatments was higher than that in CK, with the highest activity observed in DSU+PU. On the 14th day after heading, all side deep fertilization treatments significantly increased NR activity by 2.55%~4.88% compared with CK, with DSU+PU showing a significant increase of 2.14% compared with DSU, and significant differences were observed among most treatments. The NR activity in all slow-release fertilizer treatments was higher than in DU, with DSU+PU significantly increasing NR activity by 2.26% compared with DU. On the 14th day after heading, all side deep fertilization treatments increased GS activity by 1.53%~6.86% compared with CK; however, the differences among DU, DSU, and CK were not significant. The GS activity in the combined application of slow-release N fertilizer and urea treatments was higher than in DSU, with DSU+PU showing a significant increase of 4.83% compared with DSU, although there was no significant difference between DSU+PU and DSU+U.

**Figure 10:**
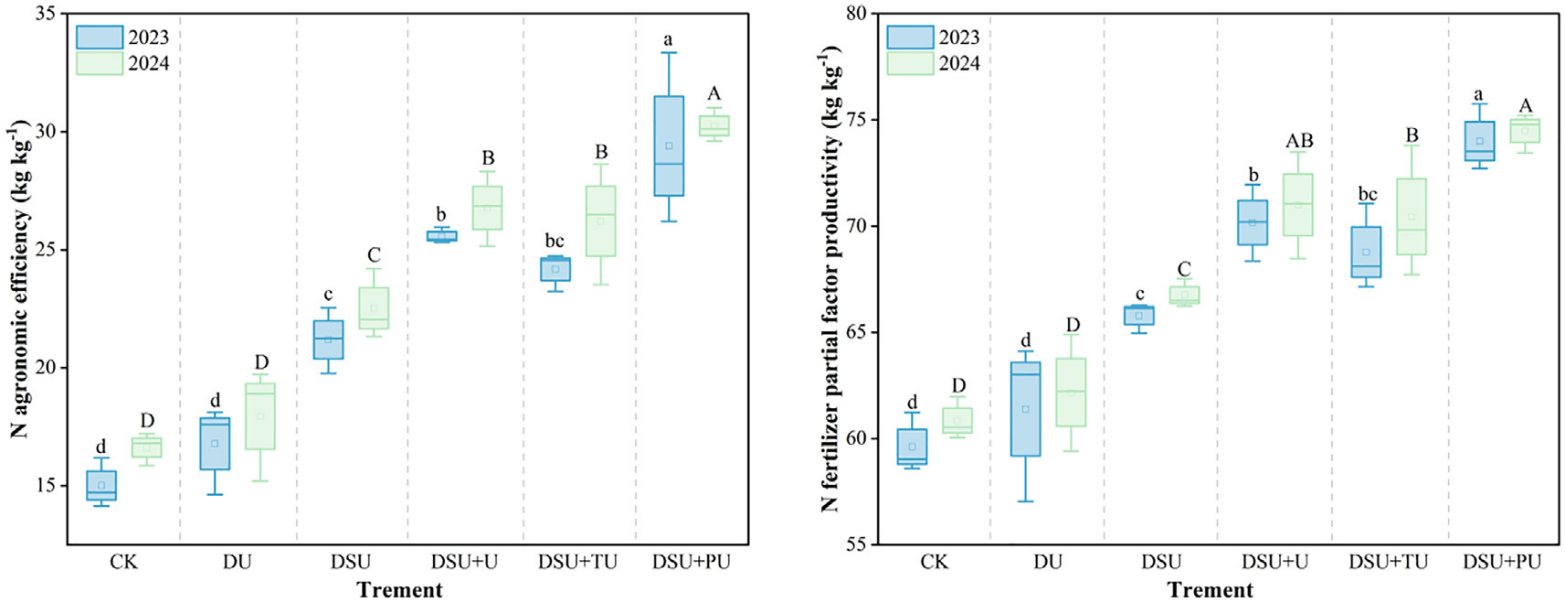
N agronomic efficiency and N fertilizer partial factor productivity of rice for different N treatments in 2023 and 2024. 0N, No N; CK, conventional fertilization control; DU, 100% urea side deep placement; DSU, 100% slow-release N fertilizer side deep placement; DSU+U, DSU combined with 30% urea applied as basal fertilizer side deep placement; DSU+TU, DSU combined with 30% urea applied as tillering fertilizer; DSU+PU, DSU combined with 30% urea applied as panicle fertilizer. Different lowercase and uppercase letters indicate significant differences at the p<0.05 level between treatments N in 2023 and 2024, respectively.

Analysis of variance showed that the year had a significant effect on NAE, while N management had a highly significant effect on NAE and NPFP (**Table 3**). Compared with CK, all side deep fertilization treatments contributed to increasing NAE and NPFP (**Figure 10**). However, the difference between DU and CK was not significant. Slow-release fertilizer treatments significantly increased NAE and NPFP by 38.35%~89.00% and 10.04%~23.27% over two years, respectively, with DSU+PU showing the highest values and significant differences from other treatments. Compared with DSU, the combined application of slow-release N fertilizer and urea treatments also enhanced NAE and NPFP, with significant increases of 15.30%~36.57% and 5.04%~12.03% over two years, respectively. Among them, DSU+PU had significantly higher NAE and NPFP than DSU+U and DSU+TU, while DSU+U showed slightly higher N use efficiency than DSU+TU, though the difference was not significant.

### 3.10 Correlation analysis

**Figure 11:**
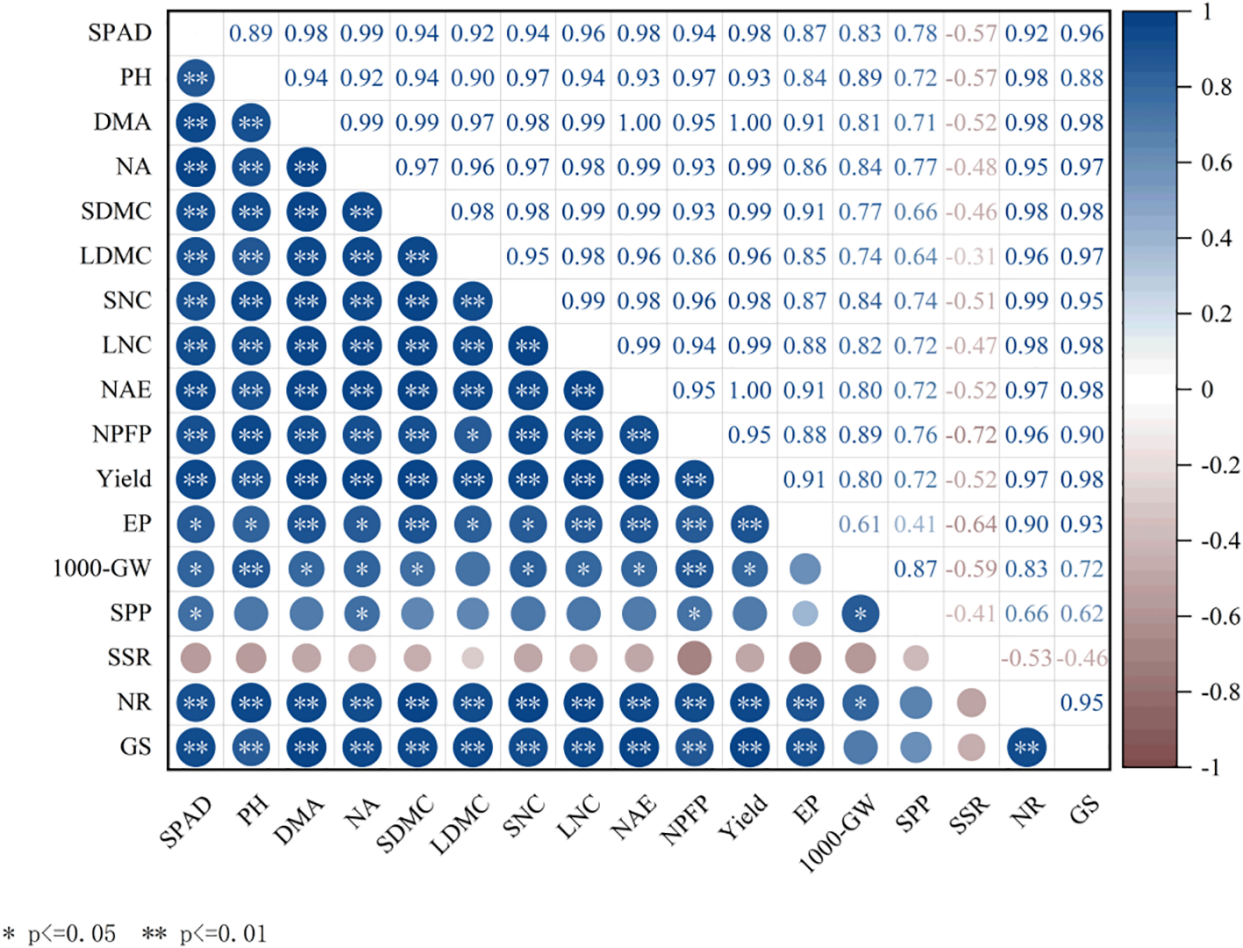
Relationship between photosynthetic material production, N accumulation and transport and yield and N use efficiency. PH, plant height; DMA, dry matter accumulationtranslocation; NA, N accumulation; SDMC, Contribution rate of stem dry matter translocation; LDMC, Contribution rate of leaf dry matter translocation; SNC, Contribution rate of stem N translocation; LNC, Contribution rate of leaf N translocation; NAE, N agronomic efficiency; NPFP, N fertilizer partial factor productivity; EP, Effective panicles; 1000-GW, 1000-grain weight; SPP, Spikelets per panicle; SSR, Seed setting rate. * and ** represent significant at 1% and 5% levels, respectively.

Correlation analysis showed that yield was significantly or highly significantly positively correlated with SPAD, PH, DMA, NA, SDMC, LDMC, SNC, LNC, AEN, PFPN, NR, GS, and other indicators (**Figure 11**). The DSU+PU treatment effectively promoted N assimilation and translocation by improving photosynthetic capacity, plant height, dry matter accumulation, N accumulation in the later growth stages, and increasing the contribution rate of stem and leaf N to the panicle after heading, as well as enhancing N metabolism enzyme activity. This ultimately led to higher yield and improved N use efficiency.

## 4 Discussion

### 4.1 Optimizing N fertilizer management increased rice yield and dry matter production

Rice dry matter accumulation and distribution form the foundation of yield formation, and post-flowering dry matter translocation is significantly positively correlated with yield (Hu et al., 2024). Compared with conventional fertilization, side deep fertilization precisely delivers nutrients to the rice root zone, effectively reducing N runoff and volatilization losses while enhancing soil nutrient retention (Wu et al., 2017). This approach promotes early and rapid tiller development, significantly increases dry matter accumulation at maturity, and enhances dry matter translocation from stems and leaves, thereby increasing the number of effective panicles and overall yield (Yao et al., 2018). Some studies have found that a single application of side deep urea can increase the leaf area index and dry matter accumulation during early growth stages but may reduce population growth rates after heading, leading to yield differences that are not significant compared to conventional fertilization (Huang et al., 2021; Zhao et al., 2021). The results of this study are consistent with those findings.

Our research shows that all side deep fertilization treatments exhibited a certain yield-enhancing effect, particularly in slow-release N treatments, which significantly increased yield compared with CK. This was primarily due to a significant increase in the number of effective panicles and seed-setting rate while maintaining stable spikelets per panicle and 1000-grain weight (**Table 4**). Additionally, side deep fertilization improved total dry matter accumulation and the efficiency of dry matter translocation from stems and leaves. Slow-release N fertilizer further reduced ineffective tillers, increased SPAD values in leaves during mid-to-late growth stages, delayed leaf senescence (**Figures 3**, **5**, **6**). However, since N release from side deep slow-release fertilizer is relatively slow, it may not fully meet rice’s bimodal N demand (Geng et al., 2015). To address this, this study applied 30% fast-acting urea in combination with side deep slow-release N, either as a basal, tillering, or panicle fertilizer. The results showed that compared to DSU, the combined application of slow-release N fertilizer and urea treatments significantly increased yield and total dry matter accumulation after heading (**Table 4**). Specifically, DSU+U and DSU+TU effectively promoted tillering, ensuring a high leaf area index during the later growth stages for sustained photosynthetic production (Wei et al., 2017). Meanwhile, DSU+PU achieved the highest yield among all treatments. This was because additional panicle fertilizer further increase rice plant height, enhanced the source-sink activity of rice, facilitating efficient dry matter translocation from vegetative organs to the panicle (Liu et al., 2015) (**Figures 3**-**6**). This process was beneficial for tiller retention, increasing the number of effective panicles, and improving grain filling (Zhu et al., 2020). In comparison, the SU treatment, which involved surface-applied slow-release fertilizer, resulted in lower nitrogen-use efficiency and grain yield than the side-deep application treatments. The lack of precise nitrogen placement in the root zone may have limited early nitrogen uptake and reduced nutrient use synchronization with crop demand (Lan et al., 2024). These results further demonstrate the importance of deep placement in enhancing nitrogen availability and improving the overall effectiveness of slow-release fertilizers.

### 4.2 Optimizing N management increased N accumulation and promoted N translocation from stems and leaves to the panicle

Nitrogen is an essential nutrient for plant growth and development, and N absorption and translocation efficiency are key factors for achieving high rice yields (Pan et al., 2017b). Compared with conventional fertilization, side deep fertilization reduces NH_4_^+^-N concentration in surface water, minimizes NH_3_ volatilization, and enhances soil fixation of NH_4_^+^-N, thereby improving N accumulation (Franciso et al., 2015). By applying N directly to the root zone, side deep fertilization promotes efficient root N uptake, increasing N accumulation and absorption rate during early growth stages while enhancing N translocation and contribution from stems and leaves (Gui et al., 2022). Additionally, slow-release N fertilizer continuously releases N, reduces urease activity during the tillering stage, delays the peak period of soil NH_4_^+^-N content to the jointing stage, ensures root vitality, and also promotes rice absorption of more N (Liao et al., 2024), which aligns with the findings of this study.

This study found that all side deep fertilization treatments contributed to increased total N accumulation and enhanced N translocation amount, output rate, and contribution rate from stems and leaves, with slow-release N treatments showing significant improvements over CK (**Figures 6**, **7**). Compared with CK, DU treatment resulted in a decline in SPAD values after heading, leading to reduced photosynthetic capacity and N accumulation in later growth stages (Jiang et al., 2014). In contrast, slow-release N fertilizer maintained photosynthetic capacity and N absorption after jointing, preventing premature senescence and enhancing N assimilation during grain filling, ultimately leading to higher yields.

Previous studies have indicated that a single application of slow-release N fertilizer may result in N deficiency in early growth stages (Gao et al., 2022) or fail to meet the critical N demand during panicle differentiation (Zhang et al., 2011). The results of this study demonstrated that, compared with DSU, the combined application of slow-release N fertilizer and urea treatments enhanced N accumulation and translocation efficiency after heading (**Figures 6**, **7**). Specifically, DSU+U and DSU+TU increased N accumulation by promoting effective tiller numbers and dry matter accumulation, while DSU+PU exhibited significantly higher N accumulation and translocation efficiency than other treatments. This is because a combination of slow-release N as a basal application and quick-release N as a panicle fertilizer compensates for the N supply deficiency in later growth stages of hybrid rice. Adequate quick-release N supply can increase root exudates and soil porosity, activating soil microbial activity, enhance photosynthetic production, promote nutrient uptake, and support plant growth and panicle development (Lv et al., 2021).

### 4.3 Optimizing N management enhanced N metabolism and N use efficiency

N absorption and utilization efficiency of rice is closely related to N metabolism, which involves key enzymes such as nitrate reductase (NR) and glutamine synthetase (GS) and is influenced by environmental factors and fertilization practices (Jin et al., 2020). Compared with conventional fertilization, side deep fertilization promotes early root development, increases NR and GS activity in flag leaves at the heading stage, enhances leaf area index and dry matter accumulation (Kargbo et al., 2016), and consequently improves NAE, NPFP, and N physiological efficiency (Cao et al., 2024). Slow-release N fertilizer further enhances N metabolism enzyme activity in rice leaves at the heading and milky stages, with the most significant effects at the heading stage, optimizing N distribution and significantly improving N use efficiency (Du et al., 2016), which aligns with the results of this study.

Our study found that peak N metabolism enzyme activity occurred 14 days after heading, during the grain-filling stage, supporting efficient N utilization and translocation. All side deep fertilization treatments enhanced NR and GS activity in flag leaves from 7 to 28 days after heading, as well as NAE and NPFP, with slow-release N treatments showing a significant increase compared with CK (**Figures 8**, **9**). This is mainly because slow-release N fertilizer stimulates urease and protease activity in the soil during the later growth stages, increases soil inorganic N and microbial biomass N content (Zhang et al., 2017c), optimizes plant N uptake and translocation. Compared with DSU, the combined application of slow-release N fertilizer and urea treatments further boosted N metabolism enzyme activity and N use efficiency, with DSU+PU showing the most significant difference from DSU. The reason may be the mutual promotion between soil inorganic N supply and soil microbial abundance, which in turn enhances leaf photosynthetic capacity and growth rate, promoting normal metabolism during the grain-filling stage. Furthermore, at 14 days after heading, NR and GS activity were significantly positively correlated with NAE, NPFP, and yield (**Figure 10**), supporting the conclusion that higher NR and GS activity in rice leaves contributes to increased yield (Sun et al., 2017). However, this study is limited to investigating the mechanism of yield increase and efficiency improvement through the aboveground parts of rice. There is a lack of systematic research on the root morphology, physiological characteristics, and soil physicochemical properties, and molecular-level studies are still limited. Additionally, this experiment was a site-specific trial, and the selected variety was a hybrid indica rice, which has a more developed root system and stronger nutrient absorption capacity compared with conventional rice. Therefore, it is necessary to conduct side deep fertilization trials combining slow-release N fertilizer and panicle urea fertilizer under different climatic conditions, soil fertility levels, and rice varieties to verify the general applicability of these results.

## 5 Conclusions

Side deep placement of slow-release N as base fertilizer combined with urea as panicle fertilizer can compensate for the N supply deficiency of slow-release N fertilizer in the later growth stages of hybrid rice. Appropriate quick-release N supply can delay leaf senescence, increase the activity of rice source-sink tissues, and ensures efficient dry matter and N translocation from vegetative organs to the panicle. This promotes normal metabolic activity during the grain-filling period, benefiting tiller retention, panicle development, and grain filling, thereby significantly improving N use efficiency and yield. At the same time, side deep application of slow-release N fertilizer combined with panicle urea fertilizer reduces the need for one less fertilization event compared with conventional fertilization, and lowers production costs compared with a single application of slow-release N fertilizer, which is conducive to large-scale production and increased economic benefits. This fertilization system represents a simplified, efficient, high-yielding approach.

## Data Availability

The original contributions presented in the study are included in the article/supplementary material. Further inquiries can be directed to the corresponding authors.
